# Diagnostic accuracy of polymerase chain reaction using conjunctival swabs for canine visceral leishmaniasis: A systematic review and meta-analysis

**DOI:** 10.1016/j.parepi.2026.e00563

**Published:** 2026-11-05

**Authors:** Marianne Rachel Domiciano Dantas Martins, Miriã Mamede Noronha de Souza, Arthur Masaharu da Nóbrega Batista, Laís Caroline Gomes Ramos, Eduardo Milton Ramos Sanchez, Lucas Rannier Ribeiro Antonino Carvalho, Ricardo Barbosa Lucena

**Affiliations:** a Postgraduate Program in Animal Science and Health (PPGCSA), Universidade Federal de Campina Grande, Patos, Paraíba, Brazil; b Social and Agricultural Sciences (CCHSA), Department of Animal Science (DSA), Universidade Federal da Paraíba, Bananeiras, Paraíba, Brazil; c Faculty of Medicine, Universidad Nacional Toribio Rodríguez de Mendoza de Amazonas, Chachapoyas, Peru; d Department of Physiology and Pharmacology – Fyfa, Karolinska Institutet, Stockholm, Sweden; e Department of Veterinary Sciences, Universidade Federal da Paraíba, Areia, Paraíba, Brazil

**Keywords:** Sensitivity, Specificity, Diagnosis, *Leishmania infantum*

## Abstract

Canine visceral leishmaniasis is an important zoonotic disease with major public health implications. Its laboratory diagnosis remains challenging, particularly in endemic areas. Polymerase chain reaction (PCR) using conjunctival swab samples has gained attention as a minimally invasive approach for the molecular detection of *Leishmania infantum*. This study aimed to synthesize the available evidence on the sensitivity and specificity of PCR using conjunctival swab samples for the diagnosis of canine visceral leishmaniasis. The systematic review was conducted according to the Preferred Reporting Items for Systematic Reviews and Meta-Analyses (PRISMA) recommendations. Searches were performed in PubMed, Web of Science, SciELO and LILACS. After the selection process, 17 studies were included in both the qualitative and quantitative syntheses. The methodological quality was assessed using the Quality Assessment of Diagnostic Accuracy Studies 2 (QUADAS-2) tool. Bivariate random-effects meta-analyses were performed to obtain pooled estimates of sensitivity and specificity, while diagnostic odds ratio (DOR) was estimated through an auxiliary univariate random-effects model. Potential small-study effects were assessed using Deeks' funnel plot asymmetry test, with a two-sided *p*-value <0.10 considered indicative of statistically significant funnel plot asymmetry. The meta-analysis yielded a pooled sensitivity of 77.8% (95% CI: 67.9 to 85.3%) and a pooled specificity of 95.5% (95% CI: 91.1 to 97.8%). While joint sensitivity and specificity estimates showed low inconsistency (*I*^2^ = 16.7%), substantial heterogeneity was observed in the DOR (*I*^2^ = 75.2%). Deeks' funnel plot asymmetry test showed no statistically significant asymmetry (*p* = 0.1765). Higher diagnostic performance tended to be observed in studies using kDNA-based protocols and bilateral conjunctival sampling. Overall, PCR using conjunctival swabs shows promising diagnostic performance under optimized laboratory conditions; however, further standardized diagnostic accuracy studies are required before this approach can be recommended as a standalone replacement for established diagnostic strategies.

## 1 Introduction

Canine visceral leishmaniasis, caused by protozoa of the genus *Leishmania*, is an important zoonosis with a wide global distribution and particular relevance in endemic regions such as Brazil (Ratzlaff et al., 2023). Diagnosis remains challenging because of the variety of *Leishmania* species involved in the etiology of the disease and the need for techniques sensitive enough to identify both symptomatic and asymptomatic dogs (Fernandes et al., 2019). Asymptomatic dogs may account for up to 85% of infected animals in some endemic areas and serve as persistent reservoirs that maintain the transmission cycle even in the absence of overt clinical disease (Paltrinieri et al., 2010; Di Muccio et al., 2012; Cavalera et al., 2022; Otranto et al., 2024). Early detection of infected dogs, including asymptomatic animals, is important because of the role of these reservoirs in maintaining disease transmission (Dantas-Torres, 2007).

The diagnostic sensitivity of molecular assays depends considerably on the type of tissue evaluated, with bone marrow, lymph node and spleen being the most suitable samples for detecting *Leishmania* DNA (Martínez et al., 2011; Latrofa et al., 2024). However, the collection of these tissues is inherently invasive, painful, and often requires sedation or specialized clinical settings, leading to poor compliance from dog owners during mass screening programs (Lombardo et al., 2012). Furthermore, sensitivity depends on the polymerase chain reaction (PCR) target, with kinetoplast DNA minicircles (kDNA) being the target most associated to high sensitivity (Solano-Gallego et al., 2017; Castelli et al., 2021). Less invasive samples, such as conjunctival swabs or blood, are alternative options in some cases in which more invasive samples cannot be obtained (Strauss-Ayali et al., 2004; Francino et al., 2006; Pilatti et al., 2009).

Conjunctival swabs have attracted interest because of their ease of collection, lower discomfort for the animal and potential application in epidemiological studies and large-scale screening of dogs (Ceccarelli et al., 2014; Leite et al., 2015; Pereira et al., 2016). PCR can detect *Leishmania* spp. infection in both clinically suspected dogs and asymptomatic dogs (Castelli et al., 2021). Unlike peripheral blood, which often shows inconsistent parasitemia and the presence of PCR inhibitors, conjunctival swab sampling allows for the detection of exfoliative epithelial cells that may harbor the parasite early in the infection process, sometimes even before seroconversion (Lombardo et al., 2012). However, available studies show wide variation in PCR sensitivity and specificity values, possibly owing to methodological differences related to the type of PCR used, the population studied, the clinical stage of the animals, the seasonal variability of disease transmission and the timing of sample collection (Cavalera et al., 2022).

Despite the growing number of studies evaluating conjunctival swab PCR, there is still no consensus on its diagnostic accuracy compared with conventional reference tests. This lack of standardization hinders the establishment of clear clinical protocols, particularly regarding the choice between conventional, nested, or real-time PCR and the most effective DNA extraction methods for low-biomass samples. Furthermore, the prognostic value of a positive conjunctival swab result in asymptomatic dogs, whether it predicts the eventual development of active disease or merely reflects transient exposure, remains a subject of debate (Cavalera et al., 2022).

The absence of a systematic synthesis of the available evidence limits the interpretation of individual results and complicates decision-making regarding the use of PCR on conjunctival swabs in clinical practice and epidemiological surveillance programs.

Therefore, this study aimed to synthesize and analyze the available evidence on the sensitivity and specificity of PCR in conjunctival swab samples for the diagnosis of canine visceral leishmaniasis, compared with other diagnostic methods described in the literature.

## 2 Materials and methods

### 2.1 Study design

This systematic review and meta-analysis adhered to the methodological guidelines of the Preferred Reporting Items for Systematic Reviews and Meta-Analyses (PRISMA). This review aimed to address the following research question: what are the sensitivity and specificity of PCR on conjunctival swab samples for the diagnosis of visceral leishmaniasis in dogs when compared with alternative diagnostic methods?

The research question was structured using the PICO strategy, comprising the following elements: P (population) – dogs suspected of having or diagnosed with visceral leishmaniasis; I (intervention) – PCR on conjunctival swab samples; C (comparison) – other diagnostic methods; and O (outcome) – diagnostic sensitivity and specificity. To ensure a robust evaluation of diagnostic accuracy, specific attention was given to the quality of the reference standards employed in the primary studies, distinguishing between high-sensitivity parasitological gold standards, such as splenic or bone marrow aspirates, and secondary methods like serology.

The study selection was conducted in accordance with PRISMA recommendations, and a flow diagram was prepared to illustrate the screening and selection process.

### 2.2 Data sources

Published studies relevant to the review topic were searched in four scientific databases: PubMed, Web of Science, SciELO and LILACS. The search was performed on October 13, 2025, without restrictions on year or language of publication. The same combination of descriptors and Boolean operators was used across all four databases: ((diagnostic AND sensitivity) OR (diagnostic AND specificity) OR diagnosis) AND (PCR OR *“*polymerase chain reaction*”*) AND conjunctival AND (swab OR samples) AND (leishmaniasis OR *“Leishmania infantum”*) AND (dog OR dogs OR canine).

All retrieved records were exported to the Mendeley Reference Manager (Elsevier, Amsterdam). Records retrieved from PubMed were exported in NBIB format and converted to a BibTeX file before being imported into the reference manager. Records retrieved from Web of Science, SciELO and LILACS were exported in RIS format and imported into the reference manager.

### 2.3 Eligibility criteria

The following inclusion criteria were established for the systematic review and meta-analysis: (1) studies including naturally infected dogs with confirmed infection, regardless of clinical presentation; (2) studies evaluating the diagnostic performance of PCR performed on conjunctival swab samples for canine visceral leishmaniasis, specifically sensitivity and specificity; (3) studies comparing PCR on conjunctival swabs with at least one alternative diagnostic method, such as serology, parasitology, or molecular assays on invasive tissues (e.g., bone marrow or spleen); and (4) observational, diagnostic accuracy, cross-sectional or cohort studies. Importantly, selected studies were required to provide sufficient primary data to allow for the extraction or calculation of diagnostic accuracy indices.

Studies were excluded based on the following hierarchical criteria: (1) non-canine populations; (2) review articles or book chapters; (3) experimental studies; (4) infection by *Leishmania* species outside the L. *infantum* complex; (5) lack of conjunctival swab samples for the index test; (6) absence of a non-infected control group; or (7) insufficient primary data to allow the extraction or calculation of sensitivity and specificity indices.

### 2.4 Study selection

The study selection was conducted in distinct stages, according to PRISMA guidelines. Following the identification and removal of duplicate entries via the reference manager, the selection of studies was divided into two main stages.

In the first stage, two reviewers independently screened titles and abstracts to filter out studies that clearly deviated from the review scope. This screening focused on identifying and excluding non-primary publications, such as review articles and book chapters, as well as studies involving non-canine populations or experimental infection models. Additionally, research focusing on *Leishmania* species outside the L. *infantum* complex or those that did not utilize conjunctival swabs for the index test were discarded during this initial phase.

In the second stage, the full texts of potentially eligible articles were comprehensively reviewed to verify their technical suitability for diagnostic accuracy synthesis. Initially, studies were discarded if full-text reading revealed that PCR was not performed on conjunctival samples, contrary to initial indications.

Subsequently, exclusions were based on the absence of a non-infected canine control group, which is essential for determining diagnostic specificity. Lastly, studies were excluded due to the lack of sufficient raw data concerning PCR performed on conjunctival swab samples to construct 2 × 2 contingency tables, preventing the calculation of sensitivity and specificity indices. Disagreements between reviewers were resolved by consensus or, when necessary, by consultation with a third reviewer.

### 2.5 Risk of bias and applicability concerns assessment

The methodological quality and risk of bias of the included studies were evaluated according to the Quality Assessment of Diagnostic Accuracy Studies-2 (QUADAS-2) tool. Prior to the assessment, review-specific guidance was developed to standardize the interpretation of the QUADAS-2 according to the objectives of this review.

Two reviewers independently assessed each included study by answering the QUADAS-2 signaling questions and assigning judgments for the risk of bias and applicability domains. Judgments that were concordant between the two reviewers were accepted directly. In cases of disagreement, the studies were independently re-evaluated by a third reviewer, whose judgment was considered final.

Risk of bias was assessed across four domains (Patient Selection, Index Test, Reference Standard and Flow and Timing). Concerns regarding applicability were assessed for the first three domains (Patient Selection, Index Test and Reference Standard). The final consensus judgments were entered into Review Manager (RevMan), version 5.4 (The Cochrane Collaboration), which was used to generate the QUADAS-2 summary bar plot and traffic light plot.

### 2.6 Data extraction

The extracted data comprised study identification (author and year), country of origin, study type, sample size and the population composition, including the presence of asymptomatic dogs, symptomatic dogs and non-infected dogs, when applicable. Furthermore, detailed information was recorded regarding the molecular method employed, the DNA extraction method, the specific molecular targets and the sampling approach. The diagnostic methods used for comparison (reference standards), sensitivity and specificity values were also systematically extracted. Data were independently extracted from the included studies by two reviewers. Any discrepancies between reviewers were assessed by a third reviewer, who made the final decision.

Each study contributed one study-level 2 × 2 contingency table (true positives, false negatives, false positives, and true negatives) to the meta-analysis. These data were derived exclusively from conjunctival samples results. To avoid statistical dependence, only one PCR result per study was retained for quantitative synthesis.

When multiple PCR assays, molecular targets, sampling strategies, or analytical approaches were used in the same study, a single index-test result was selected for quantitative synthesis according to predefined criteria. Priority was given, first, to the qualitative diagnostic approach explicitly identified by the original authors as the main or preferred assay. When no such indication was provided, priority was given to the assay most consistent with the primary objective of evaluating conjunctival swab PCR as a qualitative diagnostic test. When results were presented separately according to clinical subgroup, DNA extraction method, or individual eye, data were combined when possible to obtain a single study-level 2 × 2 contingency table. When both unilateral and bilateral conjunctival swab sampling strategies were described, the bilateral sampling result was selected because it better represents an animal-level diagnostic strategy and reduces the risk of classifying infection status based on a single ocular site. This decision was made to improve comparability across studies and was not based on the magnitude of the reported sensitivity or specificity estimates.

### 2.7 Data analysis

Diagnostic accuracy meta-analysis was synthesized using a bivariate random-effects meta-analysis based on the Reitsma model using the R package mada (version 0.5.12). This model jointly estimates pooled sensitivity and specificity while accounting for the correlation between these measures across studies. The fitted bivariate model was also used to generate the summary receiver operating characteristic (SROC) curve, estimate the area under the curve (AUC) and calculate the normalized partial area under the curve (pAUC). Additionally, between-study heterogeneity was quantified within the Reitsma framework, incorporating the specific measures proposed by Zhou and Dendukuri and by Holling.

Using the R meta package (version 8.5.0), the pooled diagnostic odds ratio (DOR) was estimated through an auxiliary univariate random-effects meta-analysis. This analysis also provided the basis for assessing potential small-study effects via Deeks' funnel plot asymmetry test. A two-sided *p*-value <0.10 was considered indicative of statistically significant funnel plot asymmetry.

Subgroup analyses were performed according to population spectrum and reference standard to investigate potential sources of variability in diagnostic performance. Population spectrum was categorized as “broad clinical spectrum” or “restricted clinical spectrum”. Reference standards were categorized as “serology”, “serology + parasitological confirmation”, or “composite reference standard”.

All statistical analyses were performed in the R environment, version 4.6.1, using the RStudio interface.

## 3 Results

The systematic workflow for study identification, screening and eligibility is detailed in Fig. 1. The systematic search initially identified 131 studies in the databases consulted, including 49 from PubMed, 75 from Web of Science, 1 from SciELO and 6 from LILACS. After the removal of 51 duplicate entries, 80 studies were screened by title and abstract, resulting in the exclusion of 39 papers that did not meet the predefined eligibility criteria. Of the 41 studies selected for comprehensive full-text evaluation, 24 papers were excluded after detailed assessment. Ultimately, 17 original studies met all criteria and were included in this systematic review and meta-analysis.

**Fig. 1:**
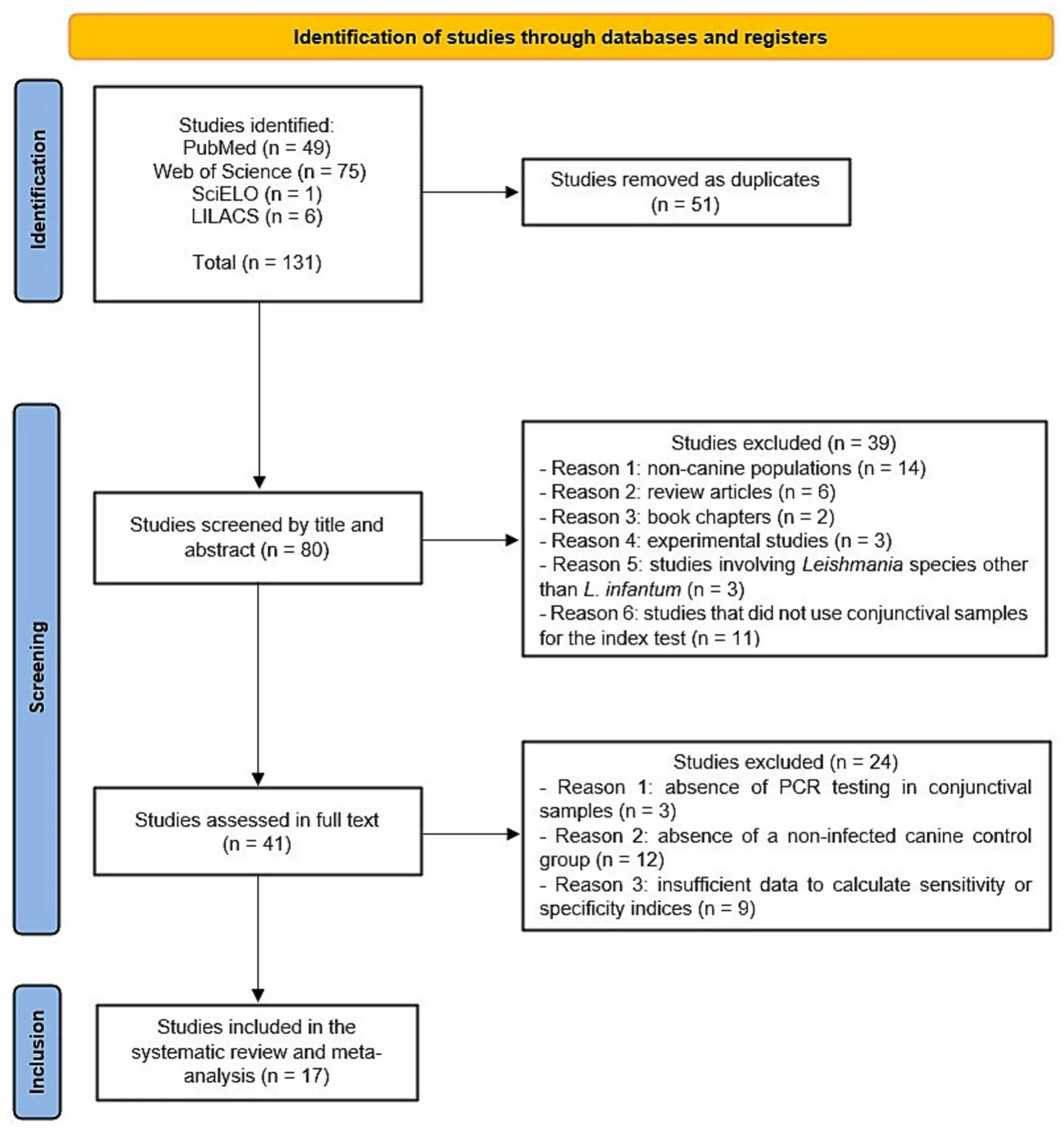
PRISMA flow diagram shows the process of inclusion and exclusion of studies in systematic review and meta-analysis.

### 3.1 Quality assessment of included studies

The risk of bias assessment showed that, in the patient selection domain, 13 studies were judged as having high risk of bias, one as having unclear risk of bias, and three as having low risk of bias. In the index test domain, one study was judged as having high risk of bias, 15 as having unclear risk of bias, and one as having low risk of bias. In the reference standard domain, two studies were judged as having high risk of bias, 12 as having unclear risk of bias, and three as having low risk of bias. In the flow and timing domain, two studies were judged as having high risk of bias, one as having unclear risk of bias, and 14 as having low risk of bias.

Regarding applicability concerns, five studies were judged as having high concern and 12 as having low concern in the patient selection domain. In the index test and reference standard domains, all 17 studies were judged as having low concern.

The overall distribution of risk of bias and applicability concerns is shown in Fig. 2, whereas the individual assessments for each included study are presented in Fig. 3.

**Fig. 2:**
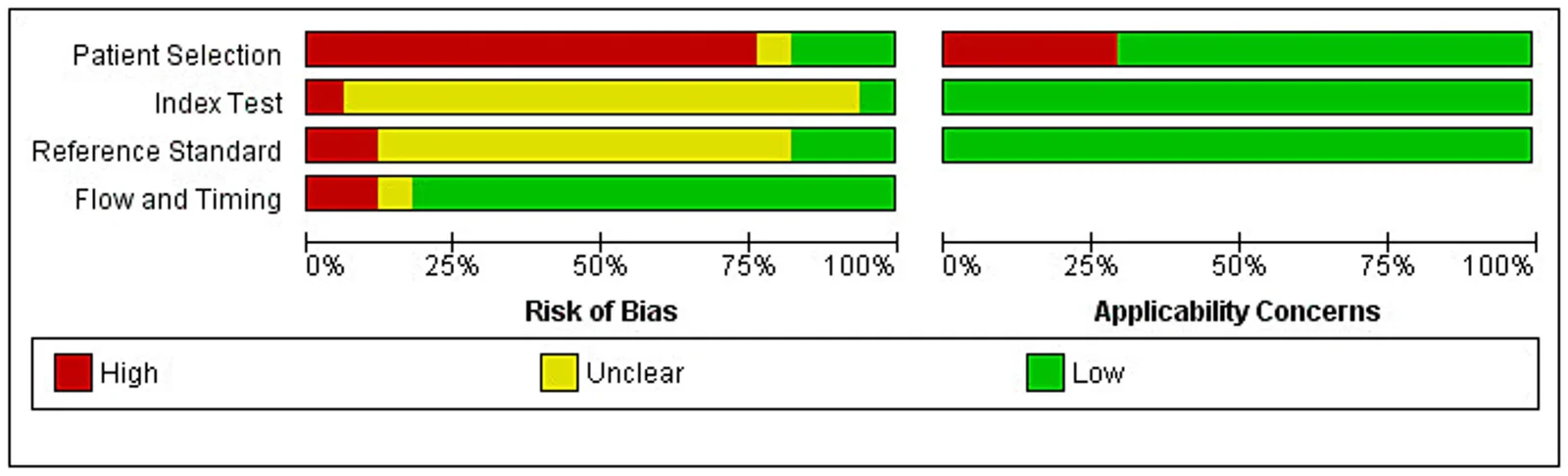
Bar graph showing the overall distribution of risk of bias and applicability concerns across QUADAS-2 domains.

**Fig. 3:**
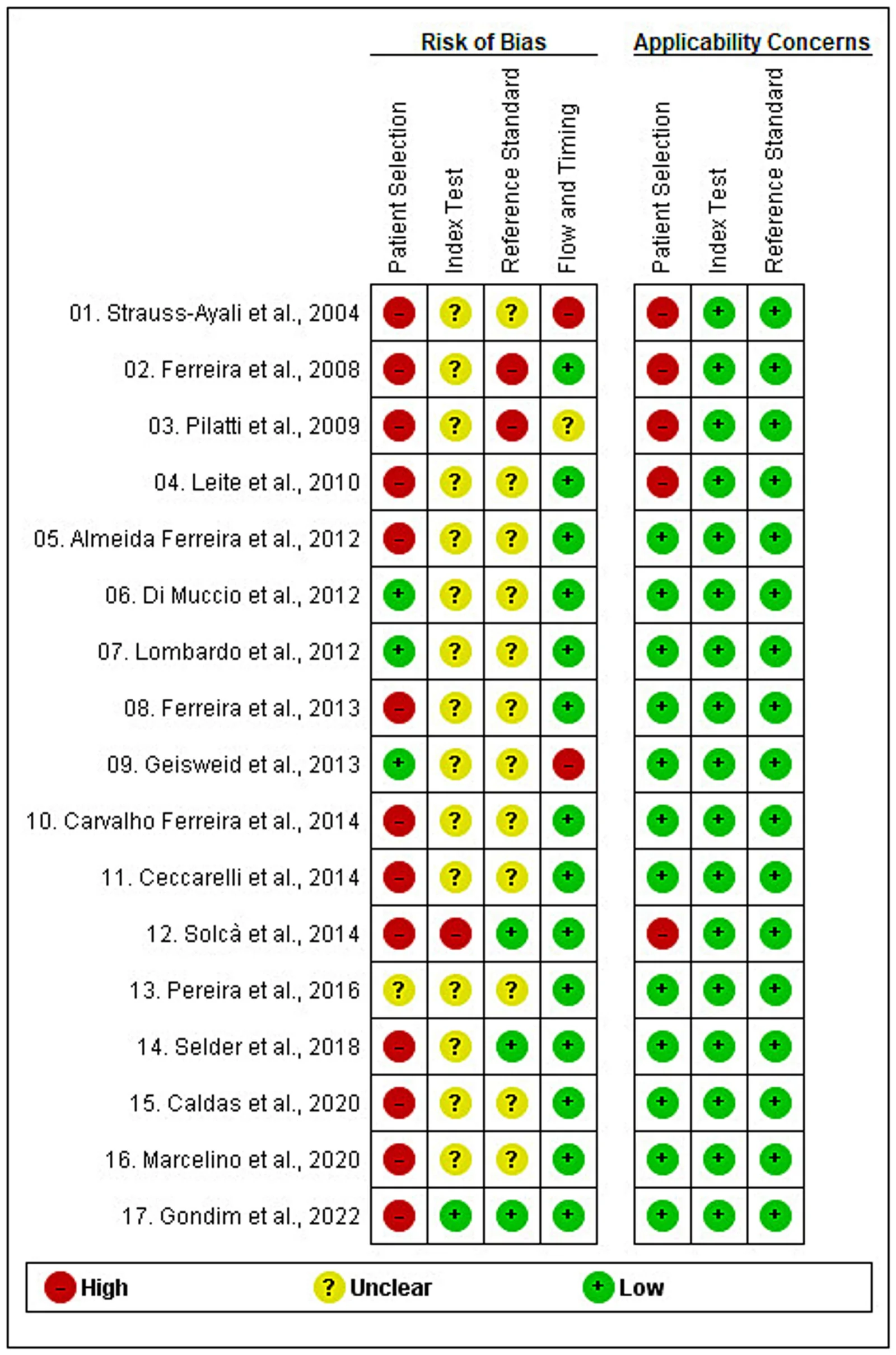
Traffic light plot of risk of bias and applicability concerns for each included study according to QUADAS-2.

### 3.2 Characterization of the included studies

The characteristics of the included studies are presented in Table 1. A predominance of studies with a cross-sectional design was observed, with only two studies including a longitudinal component.

**Table 1:** Geographical, methodological and population characteristics of the 17 studies included in the systematic review and meta-analysis.

Study (Author, year)	Country	Study type	Number of dogs	Population composition
Strauss-Ayali et al., 2004	Israel	Cross-sectional and longitudinal	98	24 symptomatic infected and 74 non-infected dogs
Ferreira et al., 2008	Brazil	Cross-sectional	56	46 symptomatic infected and 10 non-infected dogs
Pilatti et al., 2009	Brazil	Cross-sectional	33	23 symptomatic infected and 10 non-infected dogs
Leite et al., 2010	Brazil	Cross-sectional	36	30 asymptomatic infected dogs and 6 non-infected dogs
Almeida Ferreira et al., 2012	Brazil	Cross-sectional	90	40 asymptomatic infected, 40 symptomatic infected and 10 non-infected dogs
Di Muccio et al., 2012	Italy	Cross-sectional and longitudinal	253	19 asymptomatic infected, 53 symptomatic infected and 181 non-infected dogs
Lombardo et al., 2012	Italy	Cross-sectional	163	12 asymptomatic infected, 66 symptomatic infected and 85 non-infected dogs
Ferreira et al., 2013	Brazil	Cross-sectional	72	6 asymptomatic infected, 56 symptomatic infected and 10 non-infected dogs
Geisweid et al., 2013	Germany	Cross-sectional	69	15 asymptomatic infected, 22 symptomatic infected and 32 non-infected dogs
Carvalho Ferreira et al., 2014	Brazil	Cross-sectional	70	30 asymptomatic infected, 30 symptomatic infected and 10 non-infected dogs
Ceccarelli et al., 2014	Italy	Cross-sectional	80	39 infected dogs (asymptomatic and symptomatic) and 41 non-infected dogs
Solcà et al., 2014	Brazil	Cross-sectional	51	24 symptomatic infected and 27 non-infected dogs
Pereira et al., 2016	Brazil	Cross-sectional	213	14 asymptomatic infected, 15 symptomatic infected and 184 non-infected dogs
Selder et al., 2018	Germany	Cross-sectional	251	87 infected (clinical status not specified) and 164 non-infected dogs
Caldas et al., 2020	Brazil	Cross-sectional	70	11 asymptomatic infected, 49 symptomatic infected and 10 non-infected dogs
Marcelino et al., 2020	Brazil	Cross-sectional	75	65 infected (clinical status not specified) and 10 non-infected dogs
Gondim et al., 2022	Brazil	Cross-sectional	52	21 asymptomatic infected, 29 symptomatic infected and 2 non-infected dogs

The studies were conducted mainly in countries endemic for canine visceral leishmaniasis, particularly Brazil (*n* = 11), followed by Italy (*n* = 3), as well as Israel (*n* = 1) and Germany (*n* = 2). The sample size ranged from 33 to 253 dogs. All studies (*n* = 17) included animals positive and negative for infection (Table 1).

### 3.3 Molecular and comparative methods

DNA extraction methods included manual protocols, commercial kits and even simplified techniques using direct lysis or supernatant. All studies evaluated PCR performance on conjunctival swab samples, using a range of technologies, including real-time PCR (qPCR), conventional PCR (cPCR) and nested PCR (nPCR). Diagnostic performance was directly influenced by molecular targets such as kDNA, ITS-1 and SSU-rDNA. The molecular methodologies employed across the studies are summarized in Table 2.

**Table 2:** Source specimens, DNA extraction methods, PCR techniques, molecular targets and comparative tests of the 17 studies included in the systematic review and meta-analysis.

Study (Author, year)	Source specimen	DNA extraction method	PCR technique and molecular target	Reference standard strategy
Strauss-Ayali et al., 2004	CS, SP, LN, BC and SK DNA	Manual (phenol-chloroform)	cPCR targeting the ITS-1 region	Serology (ELISA), parasitological culture and PCR (SP aspirate and/or LN)
Ferreira et al., 2008	CS, BC and BL DNA	Manual (phenol-chloroform) or boiling method for CS; Commercial kit (Wizard Genomic DNA Purification Kit for BC; treatment of filter paper discs)	cPCR with hybridization targeting the kDNA minicircle (120 bp)	Serology (ELISA, IFAT and CFR)
Pilatti et al., 2009	CS DNA	Manual (phenol-chloroform)	cPCR with hybridization (targeting kDNA), seminested PCR (targeting kDNA) and nPCR (targeting ITS-1 and SSU-rDNA)	Serology (ELISA, IFAT and CFR)
Leite et al., 2010	CS, SK and BL DNA	Manual (phenol-chloroform) for CS; commercial kit (GE Healthcare) for BL; commercial kit (Promega) for SK biopsy	cPCR with hybridization targeting the kDNA minicircle (120 bp) and nPCR targeting the ITS-1 region	Serology (ELISA and IFAT) and parasitological examination of BM
Almeida Ferreira et al., 2012	CS, SK, BM and BL DNA	Manual (phenol-chloroform) for CS; commercial kit (GE Healthcare) for BL and BM; commercial kit (Promega) for SK biopsy	cPCR with hybridization targeting the kDNA minicircle (120 bp) and qPCR targeting the DNA polymerase gene (single-copy)	Serology (ELISA and IFAT) and parasitological tests (NNN culture and Giemsa-stained smears of BM and SK)
Di Muccio et al., 2012	CS, LN and BC DNA	Thermal and enzymatic lysis with direct use of the supernatant for CS; commercial kit (Invitrogen) for BC	nPCR targeting SSU-rDNA	Serology (IFAT), cytology (LN) and PCR (BC)
Lombardo et al., 2012	CS, OS, LN and BL DNA	Commercial kit (Invitrogen)	qPCR targeting the conserved region of kDNA	Serology (IFAT) and DTH test
Ferreira et al., 2013	CS, NS, OS, ES, BM and SK DNA	Manual (phenol-chloroform) for CS, NS, OS and ES; commercial kit (Macherey-Nagel) for BM and SK biopsy	cPCR targeting kDNA	Serology (ELISA and IFAT) and parasitological culture (BM)
Geisweid et al., 2013	CS, BM, LN and BL DNA	Commercial kit (Qiagen) for CS, LN and BL; manual (phenol-chloroform) for BM	qPCR targeting kDNA-120 bp	PCR of invasive tissues (BM and LN) and BL
Carvalho Ferreira et al., 2014	CS, blood, BM and SK DNA	Manual (phenol-chloroform) for CS; commercial kit (GE Healthcare) for BL and BM; commercial kit (Promega) for SK biopsy	qPCR targeting kDNA and nPCR targeting ITS-1	Serology (ELISA and IFAT) and parasitological examination (BM)
Ceccarelli et al., 2014	CS and BC DNA	Crude lysate for CS; commercial kit (Qiagen) for BC	qPCR targeting the conserved region of kDNA	Serology (IFAT)
Solcà et al., 2014	CS, SP, BM, LN, SK and BL DNA	Manual (phenol-chloroform) for CS; commercial kit (Qiagen) for SP aspirate, BM, LN, SK biopsy and BL	qPCR targeting the kDNA minicircle	Latent class analysis (LCA) integrating serology (DPP), culture (SP aspirate) and qPCR (SP)
Pereira et al., 2016	CS and BL DNA	Manual (phenol-chloroform) for CS; manual (salting-out) for BL	cPCR targeting the conserved region of kDNA	Serology (IFAT)
Selder et al., 2018	CS, BM, LN and BL DNA	Commercial kit (Qiagen) for CS, LN and BL; manual (phenol-chloroform) for BM	qPCR targeting kDNA-120 bp	PCR on multiple specimens (blood, BM, LN and CS)
Caldas et al., 2020	CS, LN, LV, SP, SK and BM DNA	Commercial kit (Qiagen)	qPCR targeting the kDNA minicircle	Serology (DPP and ELISA), direct parasitological examination (LN, SP, SK and BM), parasitological culture (BM and SP) and immunohistochemistry (SP, LV, SK and LN)
Marcelino et al., 2020	CS, BM, LN and SK DNA	Commercial kit (Qiagen)	cPCR (comparison of targets: kDNA-145 bp, kDNA-120 bp, ITS-1, hsp70–234 bp and hsp70–1300 bp)	Direct microscopy, culture and immunohistochemistry of SK, BM, SP and LN
Gondim et al., 2022	CS and BM DNA	Commercial kit (Qiagen) for CS; commercial kit (Macherey-Nagel) for BM	cPCR targeting the kDNA minicircle	PCR (CS and BM) and parasitology (BM)

Note: BC – buffy coat; BL – blood; BM – bone marrow; CFR – Complement Fixation Reaction; CS – conjunctival swab; DHT – delayed-type hypersensitivity; DPP – Dual Path Platform; ELISA – Enzyme-Linked Immunosorbent Assay; ES – ear swab; IFAT – Indirect Fluorescence Antibody Test; LN – lymph node; LV – liver; NNN – Novy-McNeal-Nicolle medium; NS – nasal swab; OS – oral swab; PCR – polymerase chain reaction; SK – skin; SP – spleen.

The reference tests showed marked diversity among the selected studies. The methodologies employed included serological tests, such as Enzyme-Linked Immunosorbent Assay (ELISA), Indirect Fluorescence Antibody Test (IFAT), as well as parasitological assays like cytology, culture and immunohistochemistry. Furthermore, several studies utilized PCR conducted on invasive tissues, such as bone marrow, lymph nodes, spleen and blood, to confirm the infection status (Table 2).

### 3.4 Sensitivity and specificity of PCR using conjunctival swabs and comparator samples

Sensitivity of PCR using conjunctival swab samples ranged from 9.2% to 100%. Values above 70% were observed in most of these studies, whereas values below this threshold were also described. In one study, sensitivity values were both above and below 70%, depending on the molecular target used. In conjunctival swab samples, specificity values ranged from 63% to 100% (Table 3).

**Table 3:** Diagnostic performance of the 17 studies included in the systematic review and meta-analysis.

Study (Author, year)	Sensitivity (CS samples)	Positivity rate (other samples)	Specificity (CS samples)	Negativity rate (other samples)
Strauss-Ayali et al., 2004	83% (unilateral) 92% (bilateral)	SP aspirate: 77.3% LN: 66.7% BC: 62.5% SK scraping: 64.7%	100%	SP aspirate: 100% LN: 100% BC: 100% SK scraping: 100%
Ferreira et al., 2008	91.3% (phenol-chloroform group) 65.2% (boiling method group) 78.3% (pooled sensitivity across both methodological groups)	BC: 21.7% BL: 30.4%	100%	BC: 100% BL: 100%
Pilatti et al., 2009	95.6% (kDNA cPCR-hybridization) 91.3% (kDNA seminested PCR) 73.9% (SSU-rDNA/ITS-1 nPCR)	Not reported	100%	Not reported
Leite et al., 2010	90% (cPCR-hybridization) 83.3% (ITS-1 nPCR)	SK biopsy: 56.7% (cPCR-hybridization) and 50% (ITS-1 nPCR) BL: 13.3% (PCR-hybridization) and 56.7% (ITS-1 nPCR)	100% (cPCR-hybridization and ITS-1 nPCR)	SK biopsy: 100% (PCR-hybridization and ITS-1 nPCR) BL: 100% (PCR-hybridization and ITS-1 nPCR)
Almeida Ferreira et al., 2012	86% (asymptomatics) 97% (symptomatics) 91.3% (pooled sensitivity across both asymptomatic and symptomatic dogs)	BM: 54% (asymptomatics) and 84% (symptomatics) SK biopsy: 51% (asymptomatics) and 78% (symptomatics) BL: 31% (asymptomatics) and 25% (symptomatics)	100% (asymptomatics) 100% (symptomatics)	BM: 93% (asymptomatics) and 78% (symptomatics) SK biopsy: 100% (asymptomatics) and 72% (symptomatics) BL: 100% (asymptomatics) and 94% (symptomatics)
Di Muccio et al., 2012	76.38%	LN: 80% BC: 50%	100%	LN: 95.7% BC: 91.2%
Lombardo et al., 2012	46.2%	LN: 43.8% OS: 15.6% BL: 11.3%	100%	LN: 82.3% OS: 93.3% BL: 96.6%
Ferreira et al., 2013	76%	BM: 90.3% SK biopsy: 80.6% NS: 87.1% OS: 78.6% ES: 42.9%	100%	BM: 100% SK biopsy: 100% NS: 100% OS: 100% ES: 100%
Geisweid et al., 2013	78.4%	BM: 89.5%	93.8%	BM: 100%
Carvalho Ferreira et al., 2014	qPCR: 96.7% (asymptomatics), 100% (symptomatics) and a98.3% (pooled sensitivity across both asymptomatic and symptomatic dogs) nPCR: 90% (asymptomatics and symptomatics)	BM: 100% (qPCR) and 96.7% (ITS-1 nPCR) SK biopsy: 96.7% (qPCR) and 73.3% (ITS-1 nPCR) BL: 90% (qPCR) and 66.7% (ITS-1 nPCR)	100%	BM: 100% SK biopsy: 100% BL: 100%
Ceccarelli et al., 2014	59%	BC: 67% (qPCR1) and 62% (qPCR2)	92,7%	BC: 92%
Solcà et al., 2014	29.2% (right CS) 37.5% (left CS)	SP aspirate: 95.8% LN: 79.2% SK biopsy: 77.3% BL: 75% BM: 50%	63%	SP aspirate: Not reported LN: 66.7% SK biopsy: 42.1% BL: 14.8% BM: 66.7%
Pereira et al., 2016	58.6%	BL: 24.1%	94%	BL: 88.5%
Selder et al., 2018	56.3%	LN: 89.5% BM: 41.2% BL: 24.9%	100%	LN: 100% BM: 100% BL: 100%
Caldas et al., 2020	66.67%	BM: 75% LN: 86.67%	100%	BM: 100% LN: 100%
Marcelino et al., 2020	92.3% (kDNA-145 bp) 66.2% (kDNA-120 bp) 55.4% (ITS-1) 20% (hsp70–234 bp) 9.2% (hsp70–1300 bp)	LN: 89.2% (kDNA-145 bp), 81.5% (kDNA-120 bp), 81.5% (ITS-1), 66.2% (hsp70–234 bp) and 53.8% (hsp70–1300 bp) BM: 83.1% (kDNA-145 bp), 68.8% (kDNA-120 bp), 71.9% (ITS-1), 58.5% (hsp70–234 bp) and 36.9% (hsp70–1300 bp) SK biopsy: 89.2% (kDNA-145 bp), 56.9% (kDNA-120 bp), 69.2% (ITS-1), 73.8% (hsp70–234 bp) and 41.5% (hsp70–1300 bp)	100%	LN: 100% (kDNA-145 bp, kDNA-120 bp, ITS-1, hsp70–234 bp and hsp70–1300 bp) BM: 100% (kDNA-145 bp, kDNA-120 bp, ITS-1, hsp70–234 bp and hsp70–1300 bp) SK biopsy: 100% (kDNA-145 bp, kDNA-120 bp, ITS-1, hsp70–234 bp and hsp70–1300 bp)
Gondim et al., 2022	91.4%	BM: 95.8% (PCR) and 13% (parasitology)	100%	BM: 100% (PCR and parasitology)

Note: BC – buffy coat; BL – blood; BM – bone marrow; CS – conjunctival swab; ES – ear swab; LN – lymph node; NS – nasal swab; OS – oral swab; SK – skin; SP – spleen.

Table 3 also summarizes the diagnostic performance of PCR using other biological samples included for comparison across the studies.

### 3.5 Quantitative synthesis

The diagnostic accuracy meta-analysis was performed using the bivariate random-effects Reitsma model with restricted maximum likelihood estimation. The pooled sensitivity was 0.778 (95% confidence interval [CI], 0.679 to 0.853), while the pooled specificity was 0.955 (95% CI, 0.911 to 0.978). Between-study heterogeneity estimated using the Zhou and Dendukuri approach was 16.7%.

The individual study sensitivity estimates ranged from 0.375 to 0.983, with substantial variation across studies (Fig. 4).

**Fig. 4:**
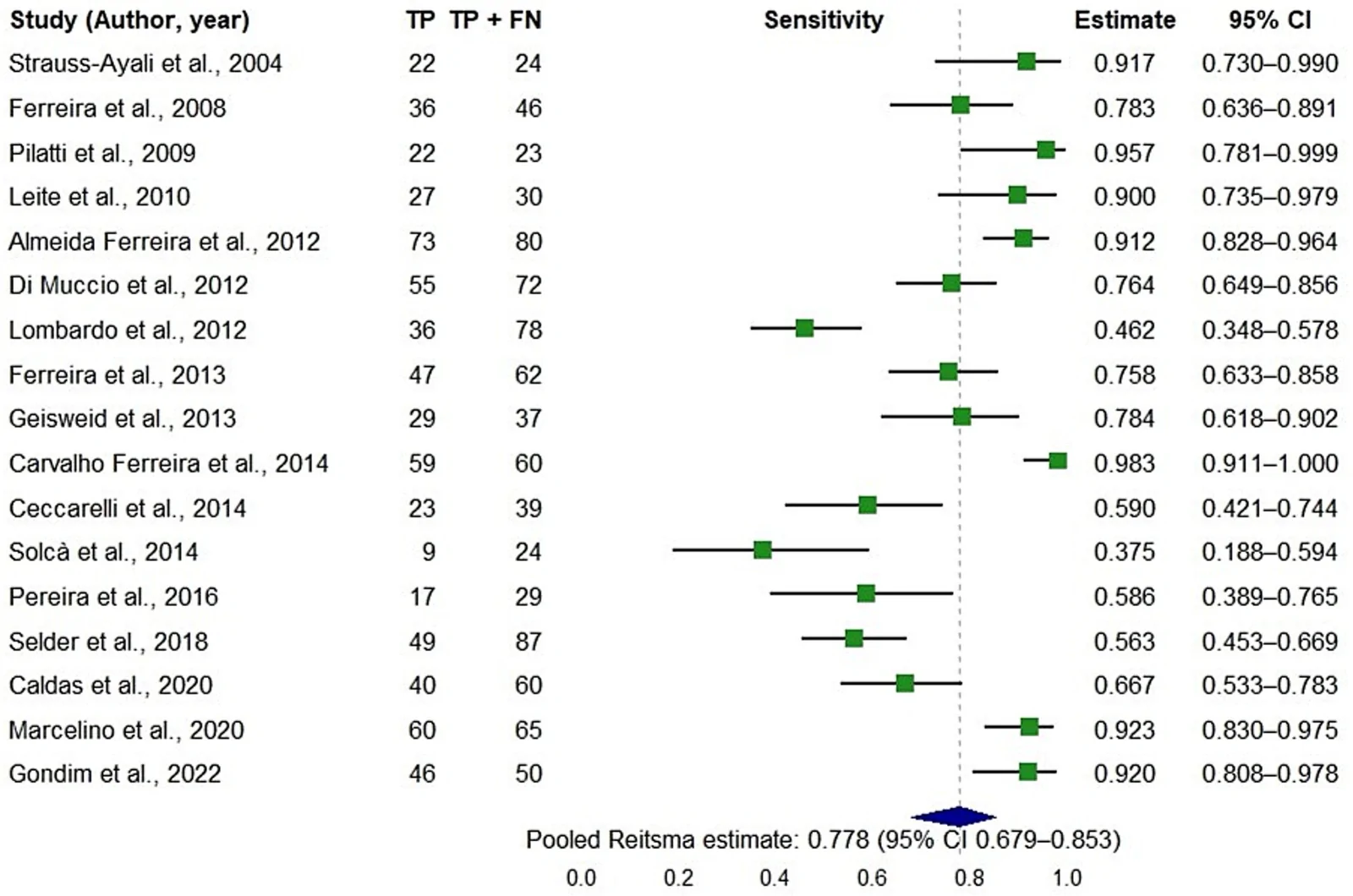
Forest plot of sensitivity estimates from the bivariate random-effects Reitsma model.

The individual study specificity estimates ranged from 0.630 to 1.000, with most studies reporting specificity of 1.000 (Fig. 5).

**Fig. 5:**
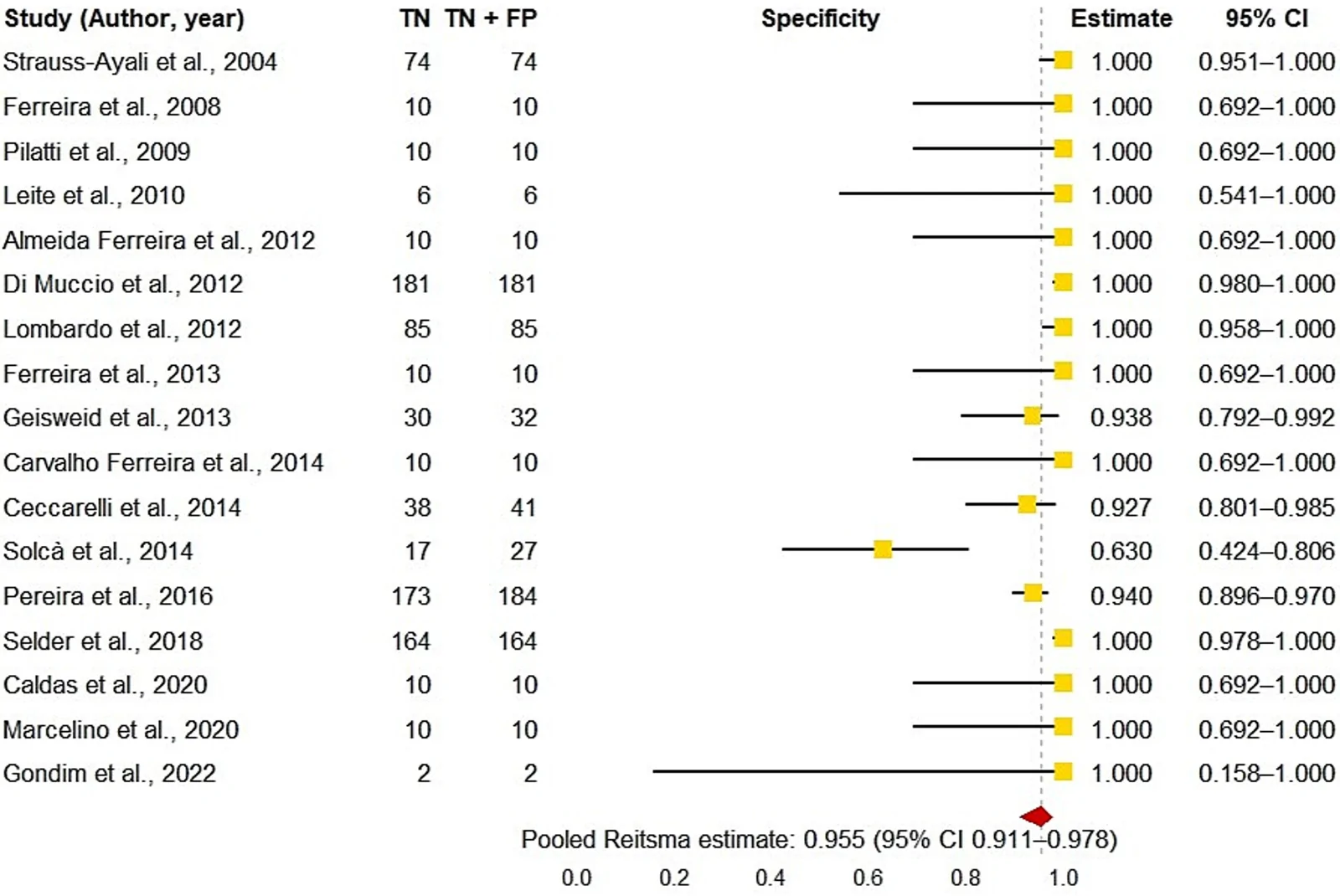
Forest plot of specificity estimates from the bivariate random-effects Reitsma model.

The overall diagnostic performance estimated by the bivariate random-effects model was summarized by the SROC curve (Fig. 6). The analysis yielded an overall area under the curve (AUC) of 0.949, with a normalized partial area under the curve (pAUC) of 0.895.

**Fig. 6:**
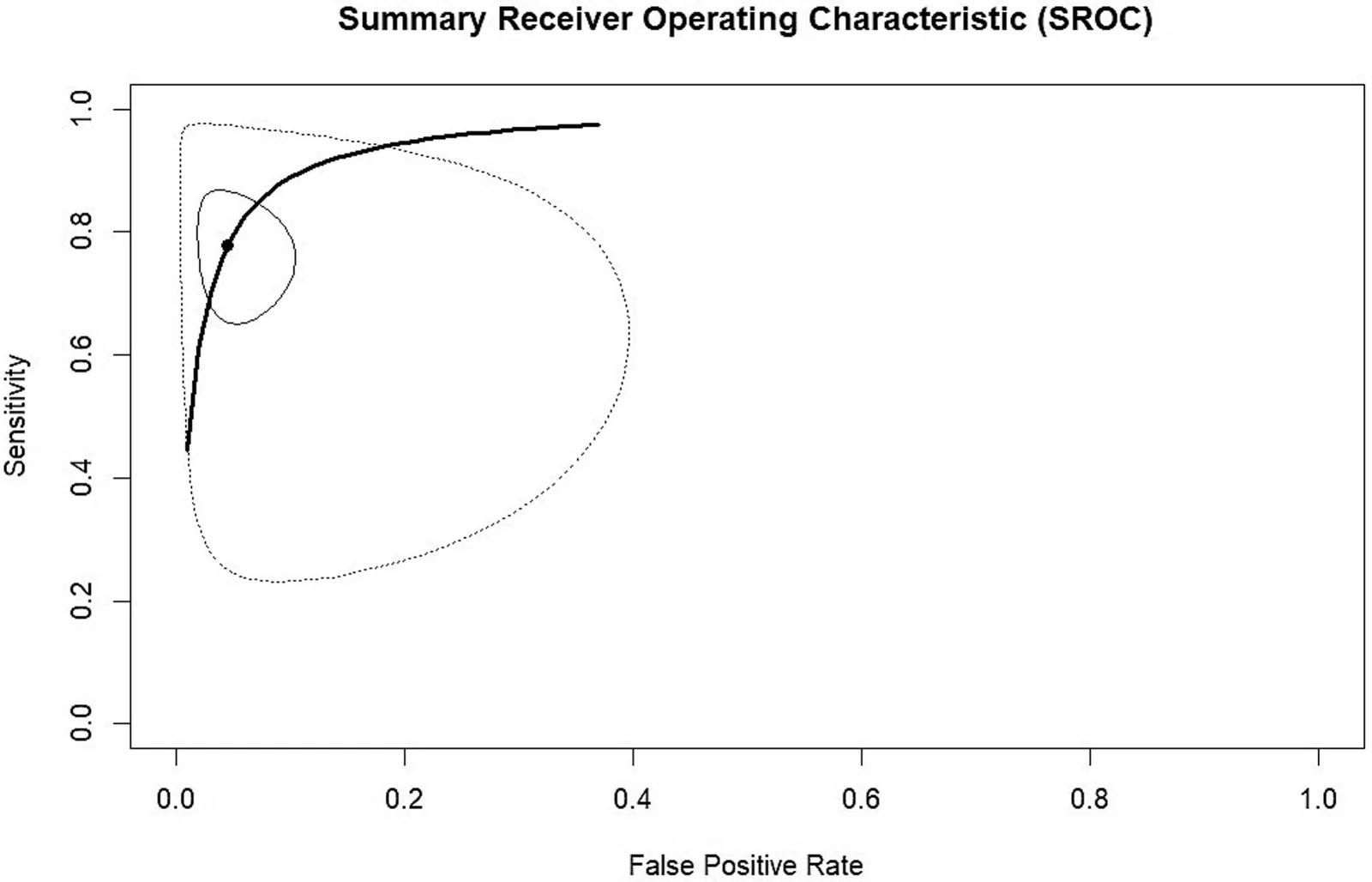
Summary receiver operating characteristic (SROC) curve obtained from the bivariate random-effects meta-analysis.

An auxiliary random-effects meta-analysis of the diagnostic odds ratio (DOR) was conducted across the 17 included studies. The pooled DOR was 76.45 (95% CI, 28.18 to 207.37). Substantial between-study heterogeneity was observed, with *τ*^2^ = 2.73, *I*^2^ = 75.2% (95% CI, 60.3% to 84.6%), and a significant Cochran's Q test (*Q* = 64.63, *df* = 16, *p* < 0.0001).

Potential small-study effects were subsequently assessed using Deeks' funnel plot asymmetry test. Study-specific DORs were plotted against the inverse square root of the effective sample size, with the vertical dashed line representing the pooled DOR (Fig. 7). Deeks' test did not indicate statistically significant funnel plot asymmetry (*t* = −1.42, *df* = 15, *p* = 0.1765), according to the prespecified significance threshold of *p* < 0.10.

**Fig. 7:**
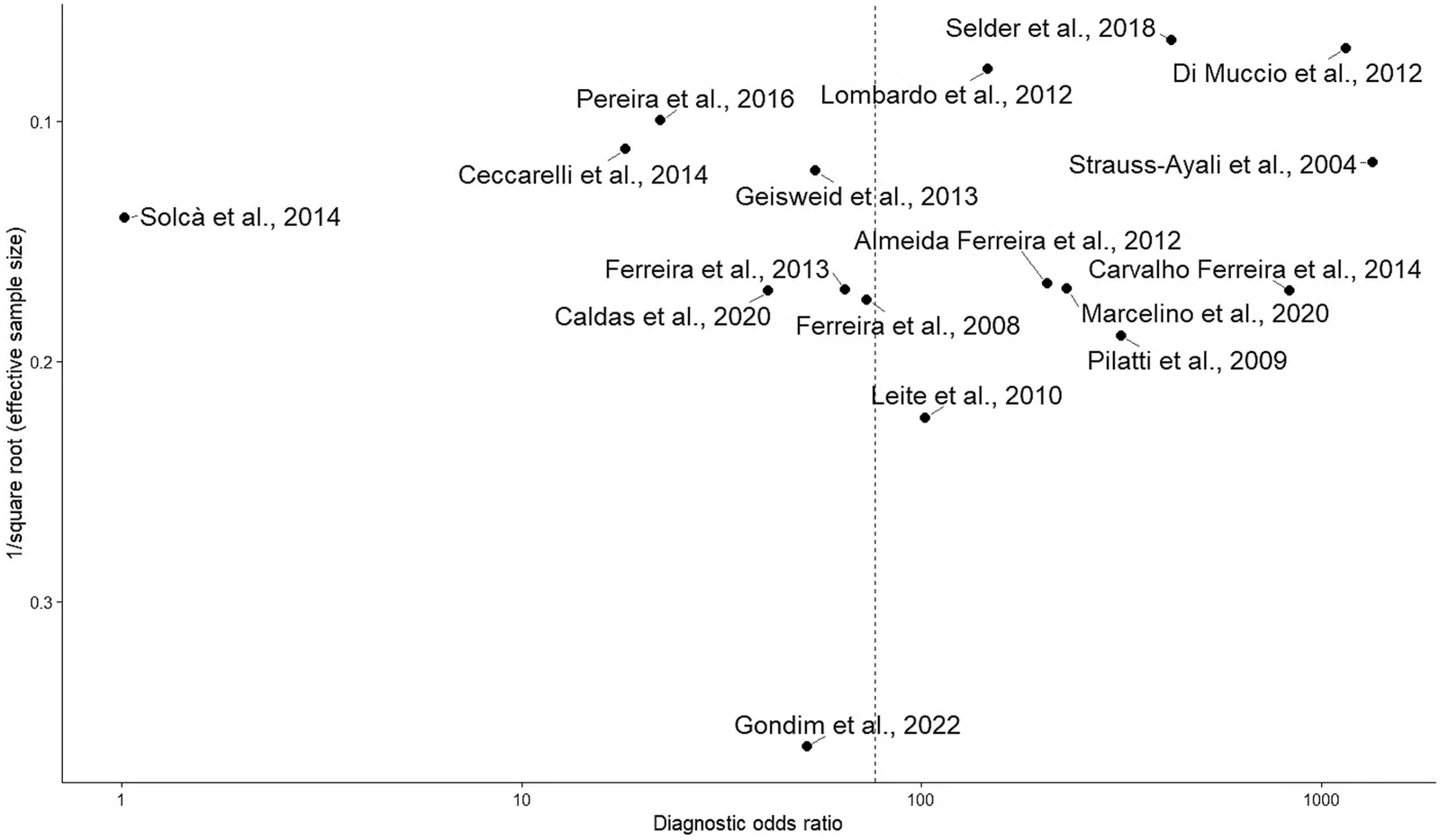
Deeks' funnel plot asymmetry test. Each point represents one of the 17 included studies. The vertical dashed line indicates the pooled diagnostic odds ratio (DOR) from the random-effects model (DOR = 76.45).

To investigate potential sources of variability in diagnostic performance, subgroup analyses were performed according to the clinical spectrum of the study population and the reference standard adopted for case classification.

Diagnostic performance according to the clinical spectrum of the study population is summarized in Table 4. Studies including a broad clinical spectrum (*n* = 12) yielded pooled sensitivity and specificity estimates of 0.769 (95% CI, 0.652 to 0.856) and 0.948 (95% CI, 0.92 to 0.966), respectively, with an AUC of 0.953. Corresponding estimates for studies with a restricted clinical spectrum (*n* = 5) were 0.808 (95% CI, 0.578 to 0.928) and 0.939 (95% CI, 0.774 to 0.986), respectively, with an AUC of 0.940. According to the Zhou and Dendukuri approach, the estimated *I*^2^ was 0% in both subgroups.

**Table 4:** Subgroup analysis according to the clinical spectrum of the study population. The “broad clinical spectrum” subgroup included Almeida Ferreira et al. (2012), Di Muccio et al. (2012), Lombardo et al. (2012), Ferreira et al. (2013), Geisweid et al. (2013), Carvalho Ferreira et al. (2014), Ceccarelli et al. (2014), Pereira et al. (2016), Selder et al. (2018), Caldas et al. (2020), Marcelino et al. (2020) and Gondim et al. (2022). The “restricted clinical spectrum” subgroup included Strauss-Ayali et al. (2004), Ferreira et al. (2008), Pilatti et al. (2009), Leite et al. (2010) and Solcà et al. (2014).

Subgroup	Pooled sensitivity (95% CI)	Pooled specificity (95% CI)	AUC	*I*^2^
Broad clinical spectrum (*n* = 12)	0.769 (0.652 to 0.856)	0.948 (0.920 to 0.966)	0.953	0%
Restricted clinical spectrum (*n* = 5)	0.808 (0.578 to 0.928)	0.939 (0.774 to 0.986)	0.940	0%

Note: AUC – area under the curve; CI – confidence interval; *I*^2^ – inconsistency statistic.

Diagnostic performance according to the reference standard adopted for case classification is summarized in Table 5. Studies using serological methods alone yielded a pooled sensitivity of 0.722

**Table 5:** Subgroup analysis according to the reference standard adopted for case classification. The “serology” subgroup included Ferreira et al. (2008), Pilatti et al. (2009), Ceccarelli et al. (2014) and Pereira et al. (2016). The “serology + parasitological confirmation” subgroup included Leite et al. (2010), Almeida Ferreira et al. (2012), Ferreira et al. (2013), Carvalho Ferreira et al. (2014) and Caldas et al. (2020). The “composite reference standard” subgroup included Strauss-Ayali et al. (2004), Di Muccio et al. (2012), Lombardo et al. (2012), Geisweid et al. (2013), Solcà et al. (2014), Selder et al. (2018), Marcelino et al. (2020) and Gondim et al. (2022).

Subgroup	Pooled sensitivity (95% CI)	Pooled specificity (95% CI)	AUC	*I*^2^
Serology (*n* = 4)	0.722 (0.532 to 0.856)	0.943 (0.906 to 0.966)	0.949	0.0%
Serology + parasitological confirmation (*n* = 5)	0.858 (0.707 to 0.938)	0.950 (0.843 to 0.986)	0.953	0.0%
Composite reference standard (*n* = 8)	0.742 (0.566 to 0.864)	0.971 (0.881 to 0.993)	0.930	35.9%

Note: AUC – area under the curve; CI – confidence interval; *I*^2^ – inconsistency statistic.

(95% CI, 0.532 to 0.856) and a pooled specificity of 0.943 (95% CI, 0.906 to 0.966), with an AUC of 0.949. Studies combining serological and parasitological confirmation yielded a pooled sensitivity of 0.858 (95% CI, 0.707 to 0.938) and a pooled specificity of 0.950 (95% CI, 0.843 to 0.986), with an AUC of 0.953. Studies using composite reference standards yielded a pooled sensitivity of 0.742 (95% CI, 0.566 to 0.864) and a pooled specificity of 0.971 (95% CI, 0.881 to 0.993), with an AUC of 0.930. According to the Zhou and Dendukuri approach, the estimated *I*^2^ was 0% for the “serology” and “serology + parasitological confirmation” subgroups, and 35.9% for the composite reference standard subgroup.

## 4 Discussion

The predominance of studies conducted in Brazil reflects the endemicity of canine visceral leishmaniasis in the country, where domestic dogs are the main reservoir of *Leishmania infantum* (Caldas et al., 2020; Marcelino et al., 2020). Studies conducted in Mediterranean foci, including Italy and Israel, also demonstrate the presence of endemic foci of the disease in these countries (Strauss-Ayali et al., 2004; Di Muccio et al., 2012; Lombardo et al., 2012; Ceccarelli et al., 2014). Even in a study conducted in Germany, a non-endemic area, qPCR on conjunctival swabs was useful to rule out infection in dogs with a history of exposure to risk areas (Geisweid et al., 2013).

The QUADAS-2 assessment revealed that five studies showed high risk of bias and high applicability concerns simultaneously in patient selection (Strauss-Ayali et al., 2004; Ferreira et al., 2008; Pilatti et al., 2009; Leite et al., 2010; Solcà et al., 2014). These studies utilized a two-gate design that compared healthy controls against infected groups restricted to either only symptomatic dogs (Strauss-Ayali et al., 2004; Ferreira et al., 2008; Pilatti et al., 2009; Solcà et al., 2014) or exclusively asymptomatic animals (Leite et al., 2010). This approach results in spectrum bias as it bypasses the full clinical range of the disease by deliberately excluding animals in intermediate or contrasting infection stages. As a result, these studies may have produced diagnostic accuracy estimates that do not generalize to clinical practice, where the primary challenge is identifying infection in dogs that truly represent the clinical population before the infection status is known.

Also regarding patient selection, a high risk of bias extended to eight other included studies (Almeida Ferreira et al., 2012; Ferreira et al., 2013; Carvalho Ferreira et al., 2014; Ceccarelli et al., 2014; Selder et al., 2018; Caldas et al., 2020; Marcelino et al., 2020; Gondim et al., 2022). The primary methodological flaw identified was the lack of consecutive or random enrollment, as all these studies employed some form of convenience sampling. For instance, in three of these studies, the dogs were deliberately recruited because they already had prior positive results from serological or parasitological tests (Almeida Ferreira et al., 2012; Caldas et al., 2020; Marcelino et al., 2020). Another methodological issue was observed in one study, which utilized a non-probabilistic sample where inclusion depended on the voluntary participation of owners (Gondim et al., 2022). These limitations suggest that the reported diagnostic accuracies may be overestimated.

The reported sensitivity of conjunctival swab PCR may have been primarily influenced by population spectrum bias. This is supported by three studies that pre-selected populations of symptomatic dogs with high parasite loads consequently achieved high sensitivities, ranging from 73.9% to 95.6% (Strauss-Ayali et al., 2004; Ferreira et al., 2008; Pilatti et al., 2009). Furthermore, one study achieved high sensitivities (90% to 100%) likely because the sample was limited to parasitologically confirmed animals, thereby excluding dogs with mild or early-stage infections (Carvalho Ferreira et al., 2014).

The only study judged to have a high risk of bias for the index test was Solcà et al. (2014), because the positivity threshold for the qPCR was not pre-specified. Instead, the authors established the cutoff using a ROC curve derived from their own data to optimize performance, which introduces a significant risk of overestimating accuracy.

Conjunctival swab collection usually results in discrepant findings when each eye is analyzed separately. The comparison between unilateral and bilateral conjunctival swab collection shows that the latter strategy increases parasite detection. This difference is reinforced by the findings of Solcà et al. (2014), who reported distinct sensitivities for qPCR in left conjunctival swabs (37.5%) and right conjunctival swabs (29.2%). Also, in the study by Strauss-Ayali et al. (2004), the sensitivity of cPCR in conjunctival swabs increased from 83% with unilateral sampling to 92% with bilateral sampling. These findings indicate that parasite distribution in the conjunctival mucosa may be uneven between the eyes of the same animal, which may lead to underdetection of infection.

High PCR sensitivity in conjunctival swabs is strongly associated with kDNA as a molecular target because of its high multicopy nature. This is corroborated by Marcelino et al. (2020), who revealed that kDNA targets exhibit superior diagnostic sensitivity for cPCR in conjunctival swabs compared to ribosomal markers like ITS-1 or single-copy genes such as hsp70.

nPCR is efficient for detecting *Leishmania* DNA when ribosomal targets are utilized. A study achieved a sensitivity of 76.38% for nPCR on conjunctival swabs using the ribosomal SSU-rDNA target (Di Muccio et al., 2012). This performance is consistent with findings from two studies that used nPCR on conjunctival swabs targeting the ITS-1 region, which reported sensitivities ranging from 83.3% (Leite et al., 2010) to 90% (Carvalho Ferreira et al., 2014).

Regarding the reference standard, the studies by Ferreira et al. (2008) and Pilatti et al. (2009) were assigned a high risk of bias. This study relied solely on serological methods, such as ELISA, IFAT and the Complement Fixation Reaction (CFR) to define the infection status of the symptomatic dogs. However, utilizing only methods with recognized limitations for confirming infection, without confirmatory parasitological or molecular testing, is insufficient to correctly classify the target condition. This reliance on an imperfect reference standard potentially leads to the misclassification of animals, which can distort the sensitivity and specificity of the conjunctival swab PCR.

The assessment of participant flow and timing revealed a high risk of bias in two studies (Strauss-Ayali et al., 2004; Geisweid et al., 2013). In Strauss-Ayali et al. (2004), significant methodological concerns were identified because not all dogs received the parasitological reference standard, and different reference tests were applied across the study groups. This inconsistent application of tests introduces verification bias, which can compromise the validity of the findings. Similarly, the study by Geisweid et al. (2013) showed a high risk of bias due to the exclusion of five dogs from the primary diagnostic accuracy analysis because the gold standard reference test (bone marrow PCR) could not be performed. Such irregularities can lead to biased estimates, potentially distorting the calculated sensitivity and specificity of the conjunctival swab PCR.

Comparison among reference standard methods indicates that the main divergence arises when conjunctival swab PCR is compared with buffy coat PCR and serology. Ferreira et al. (2008) demonstrated that the frequency of parasite identification with cPCR on conjunctival samples can reach more than fourfold higher than on buffy coat. This is consistent with another study which found that nPCR on conjunctival swabs yielded positive results more than twice as often as on buffy coat (Di Muccio et al., 2012). Also, the relationship between positivity and clinical status suggests that blood may be a less reliable diagnostic matrix for this disease. This is aligned with a study that showed an association between conjunctival swab cPCR positivity and the presence of clinical signs, whereas blood cPCR positivity was associated with the absence of these signs (Pereira et al., 2016).

From another perspective, the performance of conjunctival swab PCR is generally lower than that of invasive methods. Two studies evidenced a consistent hierarchy of parasite identification, with detection being least frequent in conjunctival swabs, followed by bone marrow and lymph node aspirates (Selder et al., 2018; Caldas et al., 2020). Similarly, one study observed that the frequency of parasite identification in conjunctival swabs using PCR was less than half of that observed in splenic samples (Solcà et al., 2014). These results indicate that the sensitivity of PCR on conjunctival swabs depends on parasite dissemination and distribution across different tissues of the body.

Despite its practical advantages, conjunctival swab use for the molecular diagnosis of canine visceral leishmaniasis presents significant limitations. A study found that conjunctival swab was ineffective for the early detection of *Leishmania*, with slower positive conversion rates compared to buffy coat samples (Gramiccia et al., 2010). Sampling variability is another critical challenge. The amount of collected biological material can vary drastically between different patients or between samples from the same individual (Ceccarelli et al., 2014). Diagnostic accuracy also depends on the specific PCR protocol and the selected molecular target (Leite et al., 2010; Marcelino et al., 2020). These variables hinder the universal standardization of the technique.

The pooled sensitivity of PCR performed on conjunctival swab of 77.8% and its wide variation reflect the significant impact of methodological heterogeneity. The peaks in sensitivity are attributed to the multicopy nature of kDNA. In one study that employed qPCR targeting kDNA, the sensitivities ranged from 96.7% (asymptomatics) to 100% (symptomatics) in infected dogs (Carvalho Ferreira et al., 2014). For cPCR targeting kDNA, the sensitivities ranging from 86% to 97% in four studies (Pilatti et al., 2009; Leite et al., 2010; Almeida Ferreira et al., 2012; Gondim et al., 2022). However, a determining factor for the decline in pooled sensitivity was the adoption of composite reference standards, which included PCR on invasive tissues. This choice of a stringent reference standard resulted in lower sensitivity values, reaching a maximum of 78.4%, across five studies (Di Muccio et al., 2012; Lombardo et al., 2012; Geisweid et al., 2013; Solcà et al., 2014; Selder et al., 2018).

The pooled specificity of PCR performed on conjunctival samples of 95.5% reflects the consistently high values reported across most investigations. This result was favoured by 13 studies that reported 100% specificity, even when different extraction methods, molecular techniques, genetic targets and populations were evaluated (Strauss-Ayali et al., 2004; Ferreira et al., 2008; Pilatti et al., 2009; Leite et al., 2010; Almeida Ferreira et al., 2012; Di Muccio et al., 2012; Lombardo et al., 2012; Ferreira et al., 2013; Carvalho Ferreira et al., 2014; Selder et al., 2018; Caldas et al., 2020; Marcelino et al., 2020; Gondim et al., 2022). These findings show that specificity of conjunctival swab PCR presents a low risk of false-positive for the diagnosis of canine visceral leishmaniasis.

Conversely, specificity values slightly below the pooled estimate, ranging from 92.7% to 94%, were reported in three studies (Geisweid et al., 2013; Ceccarelli et al., 2014; Pereira et al., 2016). Notably, a markedly lower specificity of 63% was estimated in a study utilizing a latent class analysis model (Solcà et al., 2014). However, this finding by Solcà et al. (2014) may have occurred because PCR detected the parasite in conjunctival swab samples from dogs that the latent class analysis considered healthy, merely because these animals were considered negative in other reference tests.

Studies with a broad clinical spectrum showed lower pooled sensitivity of PCR performed on conjunctival swab (76.9%) compared to the restricted clinical spectrum subgroup (80.8%). This pattern may reflect spectrum bias, because investigations that selectively enroll symptomatic dogs tend to inflate diagnostic accuracy. In contrast, populations including asymptomatic dogs or those in early infection stages reflect a more realistic sensitivity. This occurs because asymptomatic carriers typically harbor lower parasite biomass or exhibit lower antibody titers, making detection more challenging (Almeida Ferreira et al., 2012; Di Muccio et al., 2012).

The choice of reference standard substantially altered the perceived accuracy. Studies using serology or composite reference standards yielded the lowest pooled sensitivity of PCR performed on conjunctival samples (72.2% and 74.2%, respectively). Biologically, this indicates that the parasite disseminates to lymphoid organs before reaching detectable levels in the eye. Conjunctival swabs are therefore more suitable markers for established rather than early infection (Strauss-Ayali et al., 2004).

Although Deeks' test did not indicate statistically significant funnel plot asymmetry, a substantial between-study heterogeneity (*I*^2^ = 75.2%) was observed in the DOR analysis. The included studies differed considerably in PCR methodology, reference standards and study populations. These methodological and clinical differences may explain the observed variability in diagnostic accuracy estimates.

A limitation of this review is that it was not prospectively registered in a public protocol repository. Although all review methods are fully described in the manuscript, prospective registration would have improved methodological transparency by documenting the protocol before study selection and data extraction.

Because several studies reported more than one PCR assay, molecular target, sampling strategy, or analytical approach, a single index-test result per study had to be selected for quantitative synthesis to avoid statistical dependence. Although predefined criteria were applied, this decision may still have influenced the pooled estimates.

Although PCR technique and molecular target are recognized as important factors influencing diagnostic performance, additional subgroup analyses specifically based on these variables were not performed. After applying the predefined eligibility criteria and data extraction strategy, the distribution of studies across these categories became highly unbalanced. As a result, reliable pooled estimates could not be obtained for the least represented subgroups. Any comparison would therefore be driven mainly by study-specific characteristics rather than by differences in molecular targets or PCR techniques.

## 5 Conclusion

PCR using conjunctival swabs shows potential as a non-invasive alternative for the diagnosis of canine visceral leishmaniasis. The analyzed studies indicate that protocols using kDNA-targeted qPCR or cPCR result in higher sensitivity. Bilateral sampling further improves these results. In contrast, inappropriate selection of the technique and molecular target, as well as unilateral sampling, were associated with lower or inconsistent sensitivity values. Overall, PCR on conjunctival swabs shows high specificity, whereas sensitivity varies according to parasite load, clinical stage and the methodological strategies employed.

## Declaration of generative AI in scientific writing

During the preparation of this work, the authors used the artificial intelligence tools ChatGPT (OpenAI, USA) and NotebookLM (Google LLC, USA) to assist with language editing and improving readability. After using these tools, the authors reviewed and edited the content as needed and took full responsibility for the content of the published article.

## Protocol registration

The protocol for this systematic review was not registered.

## CRediT authorship contribution statement

**Marianne Rachel Domiciano Dantas Martins:** Writing – review & editing, Writing – original draft, Methodology, Investigation, Formal analysis, Conceptualization. **Miriã Mamede Noronha de Souza:** Writing – review & editing, Methodology, Investigation, Formal analysis. **Arthur Masaharu da Nóbrega Batista:** Writing – review & editing, Methodology, Investigation. **Laís Caroline Gomes Ramos:** Writing – review & editing, Software, Formal analysis, Data curation. **Eduardo Milton Ramos Sanchez:** Writing – review & editing, Validation, Resources, Data curation. **Lucas Rannier Ribeiro Antonino Carvalho:** Writing – review & editing, Supervision, Resources, Project administration, Investigation, Funding acquisition, Data curation. **Ricardo Barbosa Lucena:** Writing – review & editing, Writing – original draft, Validation, Supervision, Project administration, Formal analysis, Conceptualization.

## Funding

This work was supported by 10.13039/501100004047Karolinska Institutet through an institutional publication agreement. The funding covered the open access publication fees.

## Declaration of competing interest

The authors declare that there are no financial, institutional, or personal conflicts of interest that may have influenced the conduct, interpretation, or publication of this manuscript.

## Data Availability

The original contributions presented in the study are included in the article. Any further inquiries may be directed to the corresponding author.
